# Circulating blood levels of IL-6, IFN-γ, and IL-10 as potential diagnostic biomarkers in gastric cancer: a controlled study

**DOI:** 10.1186/s12885-017-3310-9

**Published:** 2017-05-30

**Authors:** Norma Sánchez-Zauco, J. Torres, Alejandro Gómez, Margarita Camorlinga-Ponce, Leopoldo Muñoz-Pérez, Ramón Herrera-Goepfert, Rafael Medrano-Guzmán, Silvia Giono-Cerezo, Carmen Maldonado-Bernal

**Affiliations:** 1 Laboratorio de Investigación en Inmunología y Proteómica, Hospital Infantil de México Federico Gómez, Dr. Márquez 162, Col. Doctores, 06720 Mexico City, Mexico; 2 División de Auxiliares de Diagnóstico y Tratamiento UMAE Hospital de Especialidades, Centro Médico Nacional-Siglo XXI, IMSSl, Avenida Cuauhtémoc 330, Col Doctores, 06720 Mexico City, Mexico; 3 Laboratorio de Bacteriología, Escuela Nacional de Ciencias Biológicas-IPN, Prolongación Manuel Carpio y Plan de Ayala, Santo Tomás, 11350 Mexico City, Mexico; 4 Unidad de Investigación en Enfermedades Infecciosas, Hospital de Pediatría, Centro Médico Nacional Siglo XXI, IMSS, Avenida Cuauhtémoc 330, Col Doctores, 06720 Mexico City, Mexico; 5 Departamento de Patología, Instituto Nacional de Cancerología, Secretaría de Salud, Av. San Fernando 22, Tlalpan, 1408 Mexico City, Mexico; 6 Departamento de Sarcomas, Tracto Digestivo Bajo, UMAE Oncología, Centro Médico Nacional Siglo XXI, IMSS, Av. Cuauhtémoc 330, Col Doctores, 06720 Mexico City, Mexico

**Keywords:** Gastric cancer, Biomarker, Inflammatory cytokines, Immunosuppressive mediators, *Helicobacter pylori*

## Abstract

**Background:**

Gastric adenocarcinoma is the third most common cause of cancer-associated death worldwide. *Helicobacter pylori* infection activates a signaling cascade that induces production of cytokines and chemokines involved in the chronic inflammatory response that drives carcinogenesis. We evaluated circulating cytokines and chemokines as potential diagnostic biomarkers for gastric cancer.

**Methods:**

We included 201 healthy controls and 162 patients with distal gastric cancer who underwent primary surgical resection between 2009 and 2012 in Mexico City. The clinical and pathological data of patients were recorded by questionnaire, and the cancer subtype was classified as intestinal or diffuse. Pathological staging of cancer was based on the tumor–node–metastasis staging system of the International Union Against Cancer. Concentrations of IL-1β, IL-6, TNF-α, IL-10, and MCP-1 in serum were measured using multiplex analyte profiling technology and concentrations of IL-8, IFN-γ, and TGF-β in plasma were measured using enzyme-linked immunosorbent assay.

**Results:**

Levels of IL-1β, IL-6, IFN-γ, and IL-10 were significantly higher and that of MCP-1 was lower in gastric cancer patients compared with controls. No differences in IL-8 or TNF-α levels were observed between gastric cancer and controls. IFN-γ and IL-10 were significantly higher in both intestinal and diffuse gastric cancer, whereas IL-1β and IL-6 were higher and TGF-β lower only in intestinal gastric cancer; MCP-1 was lower only in diffuse gastric cancer. IFN-γ and IL-10 levels were significantly higher in early (I/II) and late stage (III/IV) gastric cancer; IL-1β and IL-8 were higher and MCP-1 was lower only in late stage (IV) patients. Receiver-operating characteristic analysis showed that for diagnosis of GC, IL-6 had high specificity (0.97) and low sensitivity (0.39), IL-10 had moderate specificity (0.82) and low sensitivity (0.48), and IL-1β and IFN-γ showed low specificity (0.43 and 0.53, respectively) and moderate sensitivity (0.76 and 0.71, respectively).

**Conclusions:**

Increased levels of IL-6, IFN-γ, and IL-10 might be useful as diagnostic biomarkers for GC; however, this needs to be confirmed with larger number of patients and with control groups other than blood donors, properly age paired. IL-1β, IL-6, MCP-1, and TGF-β differentiate intestinal from diffuse GC. IFN-γ and IL-10 might be useful for diagnosis of early stage GC, and IL-1β, IL-8, and MCP-1 for late stages of the disease.

**Electronic supplementary material:**

The online version of this article (doi:10.1186/s12885-017-3310-9) contains supplementary material, which is available to authorized users.

## Background

Gastric cancer (GC) remains a major health problem worldwide, representing the third most common cause of death from all cancers [1]. Mortality rates are higher in Asian and Latin American countries, where cases are usually diagnosed at later stages, leading to very low survival rates. The infection of the gastric mucosa by *Helicobacter pylori* represents the major risk factor in over 65% of all distal GC, and recent evidence suggests that it might also play a role in proximal GC [2, 3]. *H. pylori* has the ability to colonize the human stomach and persist for decades, eliciting a chronic long-lasting inflammatory response that varies in magnitude depending on the genetic background of the host, the virulence of the *H. pylori* strain, and environmental factors [4]. The differences in the gastric inflammatory response between hosts may partially explain the different outcomes observed in *H. pylori-*infected patients [5]. Understanding the natural history of the infection may help to identify potential biomarkers for the early diagnosis of GC to allow timely treatment to reduce mortality.

The interaction of *H. pylori* with the gastric epithelium induces the production of interleukin (IL)-8, IL-6, and IL-1β. These cytokines are chemotactic for neutrophils and mononuclear cells, and their production leads to a proliferative response with a dense infiltrate of neutrophils and macrophages in the gastric mucosa, resulting in a chronic active gastritis [6]. *H. pylori* also induces gastric mucosal infiltration by dendritic cells and T and B cells, and stimulates secretion of macrophage chemotactic protein (MCP)-1, tumor necrosis factor (TNF)-α, IL-12, IL-10, transforming growth factor (TGF)-β, and interferon (IFN)-γ [5]. Among other deleterious effects that predispose cells to oncogenic transformation, the inflammatory mediators produced by this decades-long gastritis may cause DNA damage, induce proliferation, and inhibit apoptosis [7]. In addition, inflammatory cytokines and chemokines increase the expression of molecular factors such as hypoxia-inducible factor-1, vascular endothelial growth factor, L-selectin, cyclooxygenase-2, and matrix metalloproteinase together with other molecules that contribute to carcinogenesis [7]. Progress of GC can be evaluated using the tumor–node–metastasis (TNM) staging classification of malignant tumors, which is an important prognostic factor for GC [8]. Ideally, biomarkers should detect the early stages (I/II) of disease when opportunities for cure are highest.

The inflammatory mediators produced locally in the gastric mucosa may reach the blood circulation and be detected in plasma samples. In this study, we tested the hypothesis that circulating levels of inflammatory cytokines and chemokines could function as indirect indicators of tissue damage, and that their measurement might be a useful biomarker for the early detection of GC, resulting in a better long-term prognosis.

## Methods

### Study population

The study included 166 patients with GC who attended the Oncology Hospital, Centro Médico Nacional Siglo XXI, Instituto Mexicano del Seguro Social (IMSS) and the Instituto Nacional de Cancerología, Secretaría de Salud in Mexico City, and who underwent primary surgical resection between 2009 and 2012. Patients ≤18 years old, with any autoimmune disease, diabetes, cancer other than GC, or having received previous treatment for GC were excluded from the study. Informed written consent was obtained from all patients prior to enrollment in the study. The Ethics Committees of the two participating institutions, IMSS and Secretaría de Salud, approved the study. The clinical and pathological data of patients were recorded using a questionnaire, and the cancer subtype was classified as intestinal GC (IGC) or diffuse GC (DGC) based on the Lauren classification [9]. Pathological staging of cancer was according to the TNM staging system of the International Union Against Cancer American Joint Committee on Cancer 2010. The study included as control group 201 healthy adults recruited in 2011–2012 (within the same period of time as for GC cases), and who were blood donors at the Central Blood Bank, Centro Médico Nacional Siglo XXI, IMSS. GC cases and healthy controls were of the same community, with similar socioeconomic and demographic conditions.

### Blood samples

Patients were asked to provide a blood sample before surgery and prior to any cancer treatment. Blood was drawn after overnight fasting into either ethylenediaminetetraacetic acid or plain tubes. The plasma or serum samples were stored at −20 °C until tested.

### Status of *H. pylori* infection

Sera of patients were tested for antibodies against *H. pylori* whole-cell antigens and against CagA protein, using a previously validated enzyme-linked immunosorbent assay (ELISA). *H. pylori* infection was defined as positive when antibodies to whole-cell antigens and/or to CagA protein were present.

### Cytokine assays

In the first part of the study, we tested plasma samples for IFN-γ, IL-8, and TGF-β; in the second part, we tested in addition for IL-10, IL-1β, TNF-α, IL-6, and MCP-1 in serum (see Additional file 1: Table S1).

The concentration of IL-8, IFN-γ, and TGF-β in plasma samples was measured by ELISA using commercially available kits (BD™OptEIA; BD Biosciences, Rockville, MD) according to the manufacturer’s instructions. The concentration of cytokine or chemokine was calculated based on standard curves provided with the kits, and results were expressed in pg/ml.

Concentrations of IL-1β, IL-6, TNF-α, IL-10, and MCP-1 were measured with multiplex analyte profiling technology (xMAP) using the Merck-Millipore, USA HSCYTOMAG-60 K Kit (Luminex®, magnetic beads Millipore, Billerica, MA) read on MAGPIX equipment (MILLIPLEX®, Millipore). The assay kits included a standard curve and two controls (high and lower concentration) for each cytokine. For both ELISA and xMAP platforms, all samples were tested in duplicate and the average values were used in the analysis.

### Statistical analysis

Values were expressed as median with interquartile ranges, and the comparison between groups was performed using Kruskal–Wallis and Mann–Whitney *U* tests, *p* < 0.05 was considered to indicated a statistically significant result. The association of each cytokine with GC was assessed using odds ratio (OR) and 95% confidence interval (CI). The results for cytokines were adjusted for patient age and sex by multivariate logistic regression analyses. A cutoff value was determined for each cytokine using a receiver-operating characteristic (ROC) curve, (Additional file 1: Table S2) and each value was used to estimate the sensitivity, specificity, positive predictive value (PPV), and negative predictive value (NPV) with 95% CI. All statistical analyses were performed using IBM SPSS Statistics software (v. 20, IBM Corp., Armonk, NY).

## Results

The characteristics of the patients and healthy donors included in the study are described in Table 1. The study included 363 individuals, of whom 162 (44.6%) were patients with GC. The group with cancer had an average age almost twice that of the healthy donor (control) group (*p* < 0.05); the ratio of females and males was similar. The frequency of seropositivity for *H. pylori* was significantly higher in the GC group than in the control group (*p* < 0.05); *H. pylori* infection showed an OR of 2.29 (1.47–3.58) for risk of developing GC. DGC was more prevalent than IGC (*p* < 0.05), and tended to occur at a younger age than IGC. Other histological types of GC represented only 22.8% of the studied sample. Only 29% of CG cases were classified as stage I or II by TNM; the majority of cases (63%) were stage III or IV. The observed female:male ratio and frequencies of GC subtypes were consistent with the recently described epidemiology of GC in Mexico [10–12].

**Table 1:** Characteristics of the gastric cancer patients and healthy blood donors included in the study

Group	No. tested (%)	Age Average ± SD	Ratio male: female	%, *H. pylori* +
Healthy donors	201	35.2 ± 11.1	1.01	53.0
Gastric Cancer	162	62.1 ± 13.2	0.93	72.3
Subtype				
Diffuse	76 (46.9)	57.8 ± 12.6	0.68	78.4
Intestinal	49 (30.2)	60.2 ± 11.8	0.92	59.2
Mixed	30 (18.5)	63.6 ± 13.7	1.21	76.7
Other	7 (4.3)	59.6 ± 11.5	7.0	83.3

### Circulating levels of IL-1β, IL-6, IFN-γ, IL-10, and MCP-1 but not IL-8, TNF-α, or TGF-β differentiate GC patients from healthy controls

**Fig. 1:**
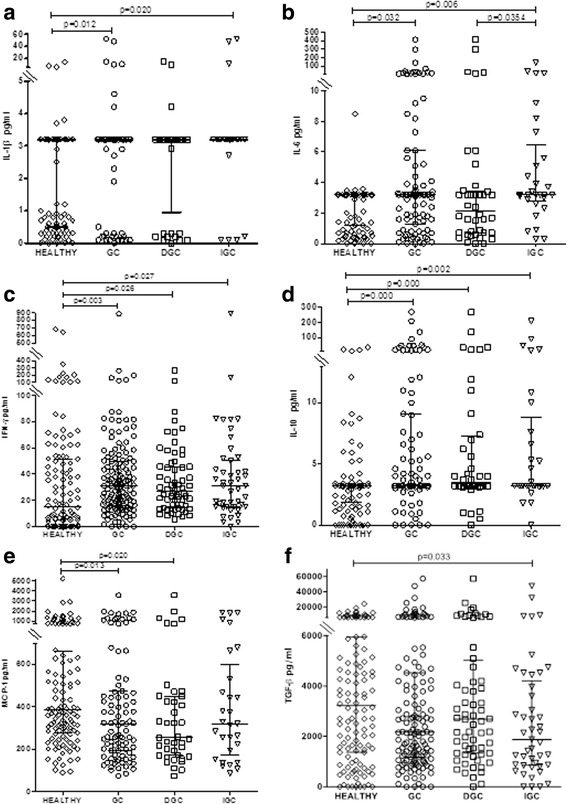
The circulating concentrations of IL-1β, IL-6, IFN-γ, IL-10, MCP-1, and TGF-β differ between healthy controls and gastric cancer patients. Circulating concentrations of IL-1β (**a**), IL-6 (**b**), IFN-γ (**c**), IL-10 (**d**), MCP-1 (**e**), and TGF-β (**f**) in healthy controls and patients with gastric cancer were measured by ELISA or xMAP. The gastric cancer group was divided into diffuse (DGC) and intestinal (IGC) types. All measurements were made in duplicate. The statistical analysis was performed by Mann–Whitney *U* test and the results for each group are presented as median with interquartile range

Circulating levels of IL-1β, IL-6, IFN-γ, and IL-10 were significantly higher and levels of MCP-1 were significantly lower in GC patients than in the healthy control group (Fig. 1). In contrast, levels of IL-8 and TNF-α did not differ significantly between GC patients and healthy controls (Additional file 2: Fig. S1). However, we noted that 22 patients in the GC group (of 143, 15.4%) had IL-8 concentrations ≥90 pg/ml, whereas in the healthy group only four individuals (of 121, 3.3%) had concentrations this high; this difference was significant (*p* = 0.0154) (Additional file 2: Fig. S1B).

The ORs for all cytokines and chemokines confirmed that elevated IL-1β, IL-6, IFN-γ, and IL-10 were risk factors for GC (Table 2).

**Table 2:** Odds ratio and *p*-values for the cytokines and chemokines evaluated as risk factor for gastric cancer

Cytokine	Positive (%)	Negative (%)	OR	IC 95%	*p*-value
IL-1β					
Healthy	65 (49)	50 (70)			
Gastric cancer	68 (51)	21 (30)	*2.49*	*1.35–4.59*	*0.003*
IL-6					
Healthy	4 (10)	111 (68)			
Gastric cáncer	35 (90)	54 (32)	*17.9*	*6.08–53.19*	*0.00*
IFN –γ					
Healthy	56 (35)	64 (61)			
Gastric cancer	101 (65)	42 (39)	*2.75*	*1.65–4.57*	*0.00*
IL-10					
Healthy	21 (36)	94 (67)			
Gastric cancer	43 (64)	46 (33)	*4.18*	*2.23–7.85*	*0.00*
MCP-1					
Healthy	59 (60)	56 (50)			
Gastric cancer	38 (40)	53 (50)	0.68	0.39–1.19	0.21
IL-8					
Healthy	49 (40)	71 (50)			
Gastric cancer	71 (60)	71 (50)	1.45	0.89–2.37	0.17
TNF-α					
Healthy	82 (57)	33 (58)			
Gastric cancer	63 (43)	28 (42)	0.91	0.49–1.65	0.76
TGF-β					
Healthy	64 (52)	56 (40)			
Gastric cancer	58 (48)	83 (60)	0.61	0.37–0.99	0.06

*p *< 0.05 statistical significance

We subsequently performed a multivariate logistic regression analysis adjusting for age and sex, and found that after adjustment IL-6, IFN-γ, IL-10, and MCP-1 had significant associations with GC (Table 3). Thus, after correcting for age, three (IL-6, IFN-γ, and IL-10) of the four cytokines associated with GC in the univariate analyses still maintained a significant association with the disease.

**Table 3:** Multivariate Logistic regression analysis for cytokines associated with risk for gastric cancer, adjusted by age and gender

Cytokines	OR	95% CI		*p*-value
		Lower	Upper	
IL-1β	1.89	0.75	*4.77*	0.178
IL-6	*8.46*	*2.04*	*35.04*	*0.003*
IFN-γ	*3.02*	*1.35*	*6.78*	*0.007*
IL-10	*5.24*	*1.95*	*14.09*	*0.001*
MCP-1	*0.33*	*0.13*	*0.81*	*0.016*

*p* < 0.05 statistical significance

### Analyses of circulating cytokines by type of GC reveal differences between IGC and DGC

We next analyzed cytokine levels in the DGC and in IGC subgroups compared with the control group, and found important differences. IFN-γ and IL-10 were significantly higher in both DGC and IGC patients (Fig. 1) than in controls. However, in contrast to the results obtained for the whole group of GC patients, we found that IL-1β and IL-6 levels were significantly higher in the IGC group but not in the DGC group, and that levels of MCP-1 were significantly lower in the DGC group but not in the IGC group. In addition, levels of TGF-β were significantly lower than controls in the IGC group but not in the DGC group (Fig. 1). These results were confirmed in a multivariate logistic regression analyses after adjusting for age and sex.

### Analyses of circulating cytokines show important differences between TNM stages of GC

**Fig. 2:**
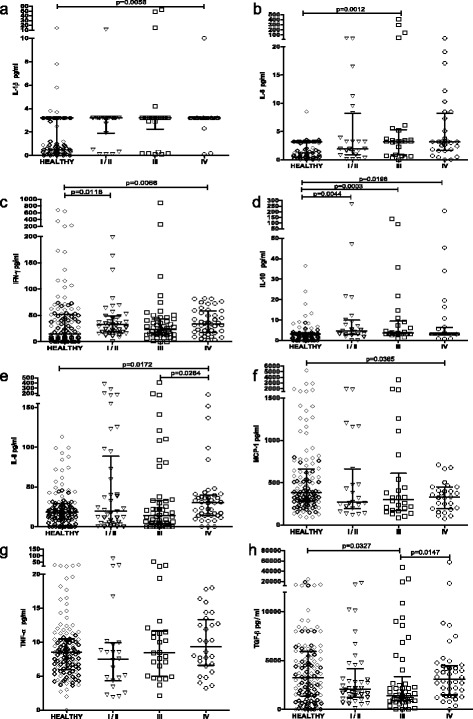
Analyses of circulating cytokines by TNM stage of GC show important differences between different stages of GC and healthy controls. Circulating levels of IL-1β (**a**), IL-6 (**b**), IFN-γ (**c**), IL-10 (**d**), IL-8 (**e**), MCP-1 (**f**), TNF-α (**g**), and TGF-β (**h**) in healthy controls and gastric cancer patients classified according to the TNM staging as stage I/II, III, or IV were measured by ELISA or xMAP. All measurements were made in duplicate. The statistical analysis was performed by Mann–Whitney *U* test and results for each group are presented as median with interquartile range

We analyzed cytokine levels in patients with different stages of GC compared with healthy controls and found that concentrations of IFN-γ and IL-10 were significantly higher in the early stages (I/II) of GC and remained higher in late stage (IV) GC (Fig. 2c and d). However, concentrations of IL-1β and IL-8 were significantly higher and that of MCP-1 significantly lower in patients with stage IV GC (Fig. 2a, e, and f) compared with controls, but not in patients with early stage GC. Levels of TNF-α were not significantly different to controls at any stage of GC (Fig. 2g). Concentrations of IL-6 were significantly higher and those of TGF-β were lower than controls in patients with late stage III GC (Fig. 2b and h). Multivariate logistic regression analyses confirmed these results after adjusting for age and sex.

### Sensitivity and specificity suggest that IL-6, IFN-γ, and IL-10 might be useful for differentiating patients with GC

Because association analyses are not sufficient to identify possible diagnostic markers, we next determined cutoff values for each cytokine using ROC analysis, which showed moderate values for the areas under the curve (described in Additional file 1: Table S2). Using these cutoff values, we explored the possible utility of these cytokines as a diagnostic test (Table 4). IL-6 showed good specificity (0.97) and low sensitivity (0.39), IFN-γ showed low specificity (0.53) and moderate sensitivity (0.71), and IL-10 showed moderate specificity (0.82) and low sensitivity (0.48), suggesting that these cytokines might be of value for identifying patients with GC. In contrast, IL-1β and MCP-1 showed poor specificity (0.43 and 0.49, respectively), eliminating them as potentially useful diagnostic biomarkers.

**Table 4:** Sensitivity and specificity estimates for each cytokine as a diagnostic test for gastric cancer

IL-1β		
Sensitivity	0.76	0.68–0.85
Specificity	0.43	0.34–0.53
PPV	0.51	0.42–0.59
NPV	0.70	0.61–0.82
IL-6		
Sensitivity	0.39	0.29–0.49
Specificity	0.97	0.93–1.00
PPV	0.90	0.80–0.99
NPV	0.67	0.60–0.74
IFN-ү		
Sensitivity	0.71	0.63–0.78
Specificity	0.53	0.44–0.62
PPV	0.64	0.57–0.72
NPV	0.60	0.51–0.70
IL-10		
Sensitivity	0.48	0.38–0.59
Specificity	0.82	0.75–0.89
PPV	0.67	0.56–0.79
NPV	0.67	0.59–0.75
MCP-1		
Sensitivity	0.42	0.32–0.52
Specificity	0.49	0.40–0.58
PPV	0.39	0.29–0.49
NPV	0.51	0.42–0.61

*PPV* positive predictive value

*NPV* negative predictive value

## Discussion

We analyzed the systemic levels of six cytokines and two chemokines associated with chronic gastric inflammation during *H. pylori* infection for potential biomarkers to identify patients with GC. We found that increased levels of IL-1β, IL-6, IFN-γ, and IL-10 and lower levels of MCP-1 significantly differentiated patients with GC from healthy controls. We also classified the GC cases according to their TNM stage and found that higher levels of IL-1β, IFN-γ, IL-10, and IL-8, and lower levels of MCP-1 differentiated late stage IV GC from healthy controls, and importantly, that IFN-γ and IL-10 differentiated patients with early stage (I/II) GC from healthy controls. These results suggest that circulating levels of cytokines may help to identify patients in the early stages of GC, offering the possibility of early detection for timely treatment.

The observed differences of these cytokines between GC patients and healthy controls are consistent with reports in both animal models and human infection. For example, IL-1β is a proinflammatory cytokine required for the efficient control of *H. pylori* infection [13]. IL-1R (−/−) mice failed to develop protective immunity but were protected against *Helicobacter*-associated gastritis and gastric preneoplasia because of their inability to generate *Helicobacter*-specific Th1 and Th17 responses [14]. In addition, the overexpression of IL-1β in the stomach of mice led to spontaneous gastric inflammation and cancer, even in the absence of *H. pylori* infection [15], and IL-1β is a potent inhibitor of gastric acid secretion [16], which may also favor the appearance of preneoplastic lesions because of hypochloridia.

The increased levels of IL-6 in GC patients and their strong association with the risk of GC that was identified in this study indicate that IL-6 plays an important role in GC, which is consistent with previous reports in animal models and in patients [16, 17]. IL-6 can upregulate DNA methyltransferases, resulting in modification of the methylation status of genes associated with tumor suppression [18]. In a mouse model of colitis-associated cancer, IL-6 stimulated the survival and proliferation of premalignant intestinal epithelial cells, mainly because of increased signal transducer and activator of transcription (STAT)3 signaling [19]. It has also been observed that IL-6 negatively affects the expression of genes associated with tumor suppression and anchorage-dependence growth in colonic epithelial cells in vitro [19]. Furthermore, it was reported that in patients with GC, expression of IL-6 and STAT3 was increased in GC tumor tissue and that the level was associated with the TNM stage of GC [20].

The higher level of IFN-γ in sera of GC patients compared with controls in this study could be indicative of its role in establishing a proinflammatory microenvironment in the gastric tissue, although its role in the promotion of GC is still controversial. IFN-γ is upregulated in the gastric mucosa after chronic *H. pylori* infection [21–24], and besides its role in the responses to bacterial infection, it has also an important tumor suppressor activity [22]. Previous studies have shown that specific T cell responses play a critical role in inducing gastric mucosal inflammation [23, 24], and suggested that IFN-γ may exacerbate gastric inflammation and favor progression to GC. However, more recent studies in a mouse model found that overexpression of IFN-γ inhibited gastric carcinogenesis induced by IL-1β and/or *Helicobacter* infection by suppressing putative gastric progenitor cell expansion and by reducing epithelial cell apoptosis via induction of an autophagy program [25]. Thus, IFN-γ is a pleiotropic mediator with both pro- and antitumorigenic activities. IL-10 is also a potent pleiotropic cytokine that has the dual ability to suppress or stimulate anticancer activity [26]. The high amount of IL-10 found in GC patients in this study would suggest that it is acting by suppressing anticancer responses. In fact, when IL-10 is elevated in blood during advanced GC, it leads to the inability to eliminate tumor cells [27, 28]. GC cells themselves can also secrete IL-10, which may explain its reduction in GC patients after surgical removal of their tumor [27, 28].

In contrast to the above cytokines, we found significantly decreased levels of MCP-1 in the sera of patients with GC. This chemokine plays an important role in the progress of *H. pylori*-related gastric diseases [29], and patients infected with *H. pylori* have significantly higher expression of *mcp-1* mRNA in the gastric mucosa than patients without *H. pylori* infection [30]. MCP-1 is a CC chemokine that is produced by gastric epithelial cells; it plays a major role in regulating migration of monocytes and lymphocytes into tissues [30] and may also promote angiogenesis and recruitment of tumor-associated macrophages [31, 32]. There are few studies reporting the circulating levels of MCP-1 in patients with GC, although one study reported significantly lower levels in cancer patients than in controls [29], which is consistent with our findings. Furthermore, the authors of that study reported that the concentration of MCP-1 in the serum of GC patients decreased in conjunction with disease progression and suggested that reduced plasma levels reflect the local consumption of MCP-1 by GC tissue [29].

We found no significant differences in the levels of circulating IL-8, TNF-α, and TGF-β between GC patients and healthy controls. The lack of significant differences in IL-8 contrasts with previous studies where circulating IL-8 levels were found to be increased significantly in patients who developed GC [33, 34]. Although we found no overall difference in IL-8 levels between the GC and healthy control groups, in GC patients there was a significantly higher frequency of IL-8 values in the upper quartile compared with controls. In addition, patients with late stage (IV) GC had significantly higher levels of circulating IL-8. These results are consistent with studies showing that IL-8 is also produced by cancer cells and may promote angiogenesis, tumor growth, tissue invasion, and metastatic spread [34, 35], and that high IL-8 expression directly correlates with a poor prognosis in GC [36]. TNF-α is one of the main mediators of inflammation and has been linked to several steps involved in carcinogenesis, including cellular transformation, survival, proliferation, invasion, angiogenesis, and metastasis [37]. However, similar to previous reports [33], we found no differences in TNF-α levels between GC patients and healthy controls. Although TGF-β can also promote tumor growth, invasion, and metastasis, it has pleiotropic activity and functions as a tumor suppressor during the early stages of GC, although it may promote tumor growth and metastasis during late stage GC [38].

We then analyzed the differences in cytokines and chemokines between the two types of GC (DGC and IGC) and found that IFN-γ and IL-10 were increased significantly in both types relative to controls. In contrast, IL-1β, IL-6, and TGF-β significantly differentiate IGC but not DGC, whereas MCP-1 was significantly lower in DGC but not in IGC. These results suggest that the pattern of cytokine production associated with GC may differentiate DGC from IGC. The lack of studies analyzing circulating cytokines in the different GC types precludes any comparison, and identifies a need for additional studies to validate our results in larger groups of patients and in different populations.

To evaluate further the utility of these markers for differentiating patients with GC from controls, we next determined the ORs and confirmed that increased levels of IL-1β, IL-6, IFN-γ, and IL-10 were all associated with increased risk of GC, noting that high levels of IL-6 increased the risk of current GC 17.9 times. The analyses also showed no increase in risk associated with MCP-1, IL-8, and TNF-α, whereas there was a trend to an association of diminished TGF-β with a decreased risk of GC. This analysis confirmed the possible value of IL-1β, IL-6, IFN-γ, and IL-10 for differentiating patients with GC in the population studied and suggested that TGF-β may also help in the identification of GC risk. We then tested the utility of these markers as a diagnostic test and found that only IL-6, IFN-γ, and IL-10 had a useful specificity (0.97, 0.53, and 0.82 respectively) and PPV (0.90, 0.64, and 0.67, respectively), but with a low sensitivity (0.39, 0.71, and 0.48, respectively). The other cytokines evaluated had poor value as a diagnostic test. Although a number of studies report the association of inflammation markers with GC, none reports the value of circulating levels of markers as a diagnostic test for GC. Such an analysis is needed for better evaluation of the utility of candidate biomarkers in GC.

In the evaluation of possible biomarkers, there is a need for consensus about the type of analyses that are required to define their utility for the timely diagnosis of patients with GC. Some authors limit their analysis to a description of the differences in candidate markers between GC and controls and report *p*-values, whereas others go further and report ORs; however, few studies test the utility of markers as a diagnostic test and report specificity, sensitivity, and predictive values. In this work, we present sequential analyses of the differences, the ORs, and diagnostic utility of circulating levels of cytokines and chemokines in patients with GC to compare the relevance of the different analytical approaches.

A consistent finding for all cytokines and chemokines tested in this study was the high level of variation between individuals in circulating cytokine levels, which, importantly, limits their possible value as candidate GC biomarkers. Another limitation of our study was that the group with GC had an average age almost twice as high as that of the healthy blood-donor control group; and although the multivariate logistic regression analyses adjusted for age confirmed association with GC, results need to be confirmed with control groups adjusted by age and sex. It is also true that despite the investment of hundreds of millions of dollars over the last 10 years in studies of cancer, none of the analyzed biomarkers has yet been approved for clinical use [39]. However, in view of the urgent need for biomarkers in GC and the lack of validated candidates, efforts to identify possible markers are badly needed, particularly in regions like Latin America that have the highest GC mortality rates. Thus, despite their poor sensitivity, our results for IL-6, IFN-γ, and IL-10 might offer a limited but still valuable test with reasonable specificity and PPV to identify patients with GC in high-risk regions.

## Conclusions

Our results suggest that high circulating levels of IL-6, IFN-γ, and IL-10 might be associated with GC, and may be potentially useful as biomarkers to identify patients at risk for GC. We also found that levels of IL-1β, IL-6, MCP-1, and TGF-β might be useful to differentiate between IGC and DGC, and that high levels of IFN-γ and IL-10 differentiated patients in the early stages of GC. Still, we caution that studies with larger groups of patients and properly matched control groups, are needed to confirm these findings. When evaluating candidate biomarkers in cancer, it is important to analyze their utility as a diagnostic test, as showed in this study.

## Additional files

Additional file 1: Table S1. Number of cases included for the different cytokines and chemokines. **Table S2.** Estimated cut off values and area under the curve for each cytokine and chemokine using a ROC analyses. (DOC 33 kb)

Additional file 2: Fig. S1. The concentrations of IL-8 and TNF-α in healthy controls and gastric cancer patients do not differ. Circulating concentrations of IL-8 (**A** and **B**) and TNF-α (**C**) in healthy donors and patients with gastric cancer were measured by xMAP. All measurements were made in duplicate. The statistical analysis was performed by Mann–Whitney *U* test and results for each group are presented as median with interquartile range. (TIFF 63 kb)

Additional file 3: Tabular data provided in format CSV. Data File. All data generated or analysed during this study are included in this file. (CSV 65 kb)

## Abbreviations

CI: Confidence interval
COX-2: Cyclooxygenase-2
DGC: Diffuse gastric cancer
EDTA: Ethylenediaminetetraacetic acid
ELISA: Enzyme-linked immunosorbent assay
GC: Gastric cancer
HIF-1: Hypoxia-inducible factor 1
IFN-γ: Interferon gamma
IGC: Intestinal gastric cancer
IL: Interleukin
MCP-1: Monocyte chemoattractant protein-1
MMP: Matrix metalloproteinase
NPV: Negative predictive value
OR: Odds ratio
PPV: Positive predictive value
ROC: Receiver-operating characteristic
STAT3: Signal transducer and activator of transcription 3
TGF: Transforming growth factor beta
TNF-α: Tumor necrosis factor alpha
TNM: Tumor–node–metastasis
VEGF: Vascular endothelial growth factor
xMAP: Multiplex analyte profiling technology
