# Correction to: Circulating blood levels of IL-6, IFN-γ, and IL-10 as potential diagnostic biomarkers in gastric cancer: a controlled study

**DOI:** 10.1186/s12885-017-3657-y

**Published:** 2017-10-02

**Authors:** Norma Sánchez-Zauco, Javier Torres, Alejandro Gómez, Margarita Camorlinga-Ponce, Leopoldo Muñoz-Pérez, Roberto Herrera-Goepfert, Rafael Medrano-Guzmán, Silvia Giono-Cerezo, Carmen Maldonado-Bernal

**Affiliations:** 1 Laboratorio de Investigación en Inmunología y Proteómica, Hospital Infantil de México Federico Gómez, Dr. Márquez 162, Col. Doctores, 06720 Mexico City, Mexico; 2 División de Auxiliares de Diagnóstico y Tratamiento UMAE Hospital de Especialidades, Centro Médico Nacional-Siglo XXI, IMSSl, Avenida Cuauhtémoc 330, Col Doctores, 06720 Mexico City, Mexico; 3 Laboratorio de Bacteriología, Escuela Nacional de Ciencias Biológicas-IPN, Prolongación Manuel Carpio y Plan de Ayala, Santo Tomás, 11350 Mexico City, Mexico; 4 Unidad de Investigación en Enfermedades Infecciosas, Hospital de Pediatría, Centro Médico Nacional Siglo XXI, IMSS, Avenida Cuauhtémoc 330, Col Doctores, 06720 Mexico City, Mexico; 5 Departamento de Patología, Instituto Nacional de Cancerología, Secretaría de Salud, Av. San Fernando 22, Tlalpan, 1408 Mexico City, Mexico; 6 Departamento de Sarcomas, Tracto Digestivo Bajo, UMAE Oncología, Centro Médico Nacional Siglo XXI, IMSS, Av. Cuauhtémoc 330, Col Doctores, 06720 Mexico City, Mexico

## Correction

After publication of the original article [1] the authors found the following author names were incorrect and needed to be amended:

- In the original article, author Javier Torres’ name was presented as J. Torres.
- In the original article author Roberto Herrera-Goepfert’s first name was incorrectly presented as Ramón.

Both these errors have been updated in the author list for this Correction. The original article has also been corrected.
