# Supplementary material for: Circulating blood levels of IL-6, IFN-γ, and IL-10 as potential diagnostic biomarkers in gastric cancer: a controlled study
Source: BMC Cancer. 2017 May 30;17:384. doi: 10.1186/s12885-017-3310-9 (PMC5450104; doi:10.1186/s12885-017-3310-9)
Supplement: Supplementary file 1 — Number of cases included for the different cytokines and chemokines. Table S2. Estimated cut off values and area under the curve for each cytokine and chemokine using a ROC analyses. (DOC 33 kb) [file 12885_2017_3310_MOESM1_ESM.doc]

**Additional file 1**

**Table S**1. Number of cases included for the different cytokines and chemokines

|  | Part 1  No. tested for IL-8, IFN-ү and TGF-β | Part 2  No. tested for IL-1β, TNF-α, IL-6, IL-10 and MCP-1 |
| --- | --- | --- |
| Healthy donors | 117 | 116 |
| Gastric cancer | 147 | 99 |
| Total | 264 | 204 |

**Table S2. Estimated cut off values and area under the curve for each cytokine and chemokine using a ROC analyses**

| **Cytokine** | **Area under the curve** | **Cut off value** |
| --- | --- | --- |
| IL-1β | 0.602 | 3.05 |
| IL-6 | 0.599 | 3.25 |
| IFN –γ | 0.612 | 18.10 |
| IL-10 | 0.662 | 3.30 |
| MCP-1 | 0.412 | 373.90 |
| IL-8 | 0.541 | 20.60 |
| TNF-α | 0.516 | 6.45 |
| TGF-β | 0.451 | 2,716.50 |
