# Supplementary figures and images for: Circulating blood levels of IL-6, IFN-γ, and IL-10 as potential diagnostic biomarkers in gastric cancer: a controlled study
Source: BMC Cancer. 2017 May 30;17:384. doi: 10.1186/s12885-017-3310-9 (PMC5450104; doi:10.1186/s12885-017-3310-9)

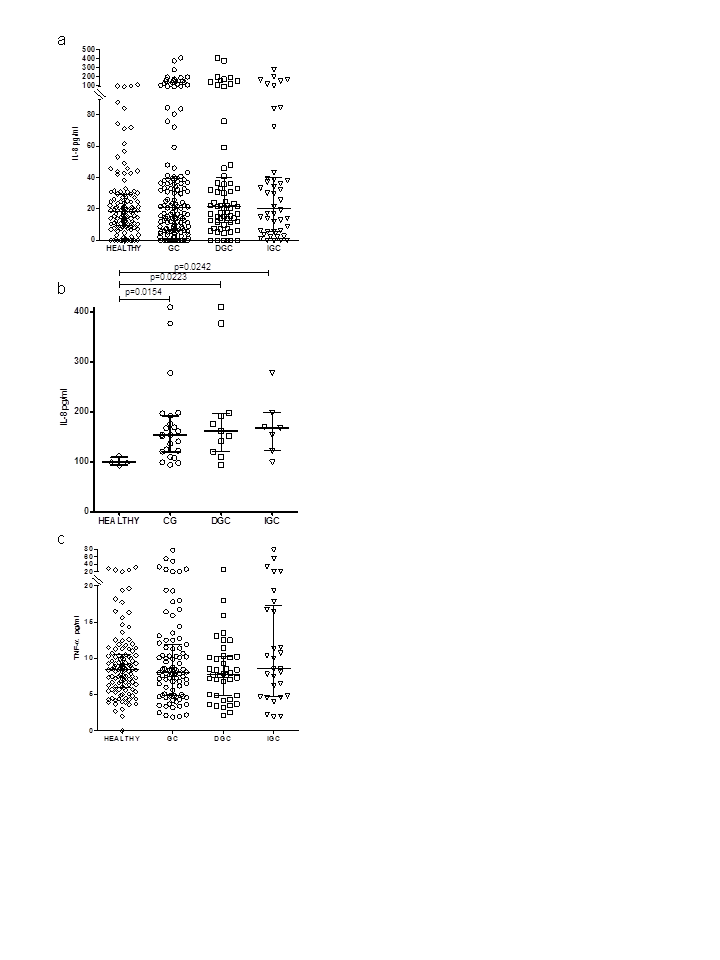

Supplement: Supplementary file 2 — The concentrations of IL-8 and TNF-α in healthy controls and gastric cancer patients do not differ. Circulating concentrations of IL-8 (A and B) and TNF-α (C) in healthy donors and patients with gastric cancer were measured by xMAP. All measurements were made in duplicate. The statistical analysis was performed by Mann–Whitney U test and results for each group are presented as median with interquartile range. (TIFF 63 kb) [file 12885_2017_3310_MOESM2_ESM.tif]
